# Sandwich-structured C/C-SiC composites fabricated by electromagnetic-coupling chemical vapor infiltration

**DOI:** 10.1038/s41598-017-13569-9

**Published:** 2017-10-13

**Authors:** Chenglong Hu, Wenhu Hong, Xiaojing Xu, Sufang Tang, Shanyi Du, Hui-Ming Cheng

**Affiliations:** 1 Institute of Metal Research, Chinese Academy of Sciences, Shenyang, 110016 PR China; 2 Center for composite materials and structures, Harbin Institute of Technology, Harbin, 150001 PR China; 3 Research and Development Center, China Academy of Launch Vehicle Technology, Bejing, 100076 PR China; 4 Shenyang National Laboratory for Materials Science, Institute of Metal Research, Chinese Academy of Sciences, Shenyang, 110016 PR China

## Abstract

Carbon fiber (CF) reinforced carbon-silicon carbide (C/C-SiC) composites are one of the most promising lightweight materials for re-entry thermal protection, rocket nozzles and brake discs applications. In this paper, a novel sandwich-structured C/C-SiC composite, containing two exterior C/SiC layers, two gradient C/C-SiC layers and a C/C core, has been designed and fabricated by two-step electromagnetic-coupling chemical vapor infiltration (E-CVI) for a 20-hour deposition time. The cross-section morphologies, interface microstructures and SiC-matrix growth characteristics and compositions of the composites were characterized by scanning electron microscopy (SEM), transmission electron microscopy (TEM) and X-ray diffraction (XRD), respectively. Microstructure characterization indicates that the SiC growth includes an initial amorphous SiC zone, a gradual crystallization of SiC and grow-up of nano-crystal, and a columnar grain region. The sandwich structure, rapid deposition rate and growth characteristics are attributed to the formation of thermal gradient and the establishment of electromagnetic field in the E-CVI process. The composite possesses low density of 1.84 g/cm^3^, high flexural strength of 325 MPa, and low linear ablation rate of 0.38 μm/s under exposure to 5-cycle oxyacetylene flame for 1000 s at ~1700 °C.

## Introduction

Carbon fiber (CF) reinforced carbon-silicon carbide (C/C-SiC) composites exhibit remarkable material properties, including low density and coefficient of thermal expansion (CTE), high strength, good thermal shock and oxidation resistance, and excellent retention of mechanical properties at high temperatures^1–4^. These qualities have made C/C-SiC composites extremely desirable for use at elevated temperature under oxidizing atmosphere, for example, re-entry thermal protection panels, rocket nozzles and brake discs^5–7^. Compared with carbon fiber reinforced carbon (C/C) composites, C/C-SiC composites exhibit better oxidation and ablation resistance due to the formation of protective silica scale over the surface, thereby improving their high-temperature performances under oxidizing atmosphere. Compared with carbon fiber reinforced silicon carbide (C/SiC) composites, C/C-SiC also present some advantages such as lighter weight, higher fabrication-time efficiency and lower component cost since carbon matrix is relatively easier to fabricate than SiC matrix.

At present, several main processes have been developed for fabrication of C/C-SiC composites, including liquid silicon infiltration (LSI), precursor infiltration and pyrolysis (PIP), slurry infiltration and hot pressing (SI-HP) and chemical vapor infiltration (CVI), etc^8–17^. In a LSI process, molten Si is used to infiltrate into a porous C/C preform and react with carbon matrix to form C/C–SiC composites at high temperatures at least over the melting point of silicon (1410 °C)^9,10^. The process has a significantly low component fabrication time and therefore, reduced component costs. However, due to the inevitable reaction between Si and carbon fibers, the mechanical properties of as-prepared composites may be damaged; as well, the matrix formed by LSI often contains free Si which limits its refractoriness and creep resistance at high temperatures. In a PIP process, polycarbosilane is infiltrated into a porous C/C and subsequent pyrolysis of the polymer is carried out at high temperatures for conversion to SiC^11,12^. The process is convenient but the major drawbacks are high shrinkage and low SiC yields, leading to the poor mechanical properties and considerable processing cycles. In a SI-HP process, ceramic slurry is infiltrated and high temperature sintering and pressing operation are required^13^. Limitations of the above process for SiC matrix are mechanical, thermal and chemical damages of carbon fibers due to high sintering temperatures and high applied pressures.

CVI is a very well established process for fabrication of C/C-SiC composites through depositing SiC matrix from gaseous precursors into porous C/C by pyrolysis of methyltrichlorosilane (CH_3_SiCl_3_, MTS) at moderate temperatures (900–1100 °C)^14,15^. This process is considered to be the most attractive one because the CVI-derived matrix has high purity, well-controlled composition, and excellent mechanical and anti-ablation properties. Another advantage of CVI is that carbon matrix and carbon fibers can be tightly wrapped by SiC matrix due to the unique growth pattern in the densification process, resulting in an effective oxidation protection of carbon. However, two factors, diffusion kinetics and surface reaction kinetics, limit the manufacturing efficiency of the conventional isothermal CVI process. In practice, the CVI tends to be controlled by surface reaction kinetics at low temperatures and pressures, whereas at high temperatures and pressures diffusion control dominates^18^. So a slow reaction rate can be desirable to ensure uniform transport of reactants throughout a porous C/C preform in order to obtain uniform matrix distribution and relatively large infiltration depth. Thus, the conventional CVI process always takes a long processing time from a few weeks to even several months to densify a porous C/C by SiC matrix.

Electromagnetic-coupling CVI (E-CVI) is an improved CVI to greatly overcome the major drawbacks of conventional CVI such as very slow deposition rate and limited infiltration depth^16,17^. Compared with the conventional CVI, the E-CVI process introduces electromagnetic field and thermal gradient into deposition space of carbon fiber preform, through directly feeding the preform with an electrical current in a cold-wall chamber, and thus partially overcomes the limitation by surface reaction kinetics and diffusion kinetics (discussed below in detail). So the process is time saving, low cost, and especially suitable for preparation of large-thickness composites. The E-CVI has been successfully used to fabricate C/C and C/SiC composites with large-thickness in a very short processing time of dozens of hours^16,17^. However, there is no investigation on the fabrication of C/C-SiC composites through depositing pyrocarbon (PyC) and SiC successively into carbon fiber fabrics. The architecture design of composites through changing distribution, composition and quantity of carbon fibers, ceramic matrices and pores is an important issue in the development of carbon fiber reinforced ceramic composites^8^. Up to now, most of C/C-SiC composites reported exhibit relatively homogeneous distribution of carbon and SiC dual matrices in macroscopic scales. However, since the C/C-SiC composites used as thermal protection materials are expected to be non-ablative for the aerospace application, SiC matrix in the core region seems to contribute less to the anti-ablation but increases the density due to its higher density than carbon. Considering that the density is a very sensitive factor for the aerospace application, it appears particularly important to design a C/C-SiC composite with SiC phase as much as possible in the surface to improve the ablation resistance but as less as possible in the core to decrease the material density. Therefore, in this paper, we designed and fabricated a sandwich-structured C/C-SiC composite (S-C/C-SiC), with two outer C/SiC layers, two gradient C/C-SiC layers and a C/C core by a two-step E-CVI process with a processing period of only 20 h. The sandwich structure, rapid deposition rate, SiC growth characteristics and material properties of the composites have been discussed.

## Methods

Needle-punched preform with a density of 0.55 g/cm^3^ was made of layers of long carbon fiber weftless plies (T700, 6k) and short-cut fiber webs; and the successive weftless plies were oriented at an angle of 90° to each other. The preform with a thickness of 9 mm was infiltrated by PyC and SiC matrices successively by a two-step E-CVI process. In the first step, the preform was firstly clamped by two graphite electrodes in a cold-wall and normal-pressure chamber. After air evacuation, the preform was directly fed with an electrical current and heated to the predetermined temperature depending on its electric resistivity. Then, the gaseous mixture of C_3_H_8_ as carbon source and Ar as diluent and protective gas was passed through the chamber. The PyC matrix was only deposited in the core of the preform since there is a temperature gradient built in the thickness direction from the core to two sides under the cold-wall effect. Through controlling temperatures in the core and surface of the preform and the deposition time, the PyC matrix gradually expanded to deposit toward the two sides. Then, a porous C/C composite with a dense core, two incompletely dense regions and two porous outer regions can be obtained in a 5 h deposition time. In the second step, the porous green C/C was subsequently infiltrated by SiC matrix using methyltrichlorosilane (CH_3_SiCl_3,_ MTS) as precursor, H_2_ as diluent gas and Ar as protective gas for 15 h. Similarly, through controlling the temperature and time, SiC preferred to deposit in the incompletely dense regions and then gradually densify the porous outer regions. Finally, a C/C-SiC composite with a sandwich structure can be obtained. For comparison, C/C and C/SiC composites were also fabricated by E-CVI technique using the same preforms.

The densities of the composites in different regions were characterized by grinding off the outer layers using a diamond grinding tool according to ref.^19^. The flexural strength of the composites was measured by three point bending test with a loading rate of 2 mm/min under a universal testing machine. The sample size was 80 mm (length) × 12 mm (width) × 8 mm (height) and the span was 70 mm. The value of the flexural strength was obtained by averaging the 5 measurements. The ablation resistance of the composites was measured by an oxyacetylene flame parallel to the axial orientation of samples with dimensions of Φ30 mm × 6 mm. The distance between the nozzle tip and the sample was 55 mm and the temperature of the sample surface reached about 1700 °C. Ablation test for each sample was performed 5 cycles for 1000 s accumulatively and the linear ablation rates were calculated after each cycle according to the change of the thickness. The value of the ablation rate was obtained by averaging the 3 measurements.

The microstructures, morphologies, and compositions of the composites were analyzed by scanning electron microscopy (SEM), transmission electron microscopy (TEM), scanning-transmission electron microscopy (STEM) combined with energy dispersive spectroscopy (EDS) and X-ray diffraction (XRD).

## Results and Discussion

### Sandwich structure and rapid deposition mechanism

**Figure 1:**
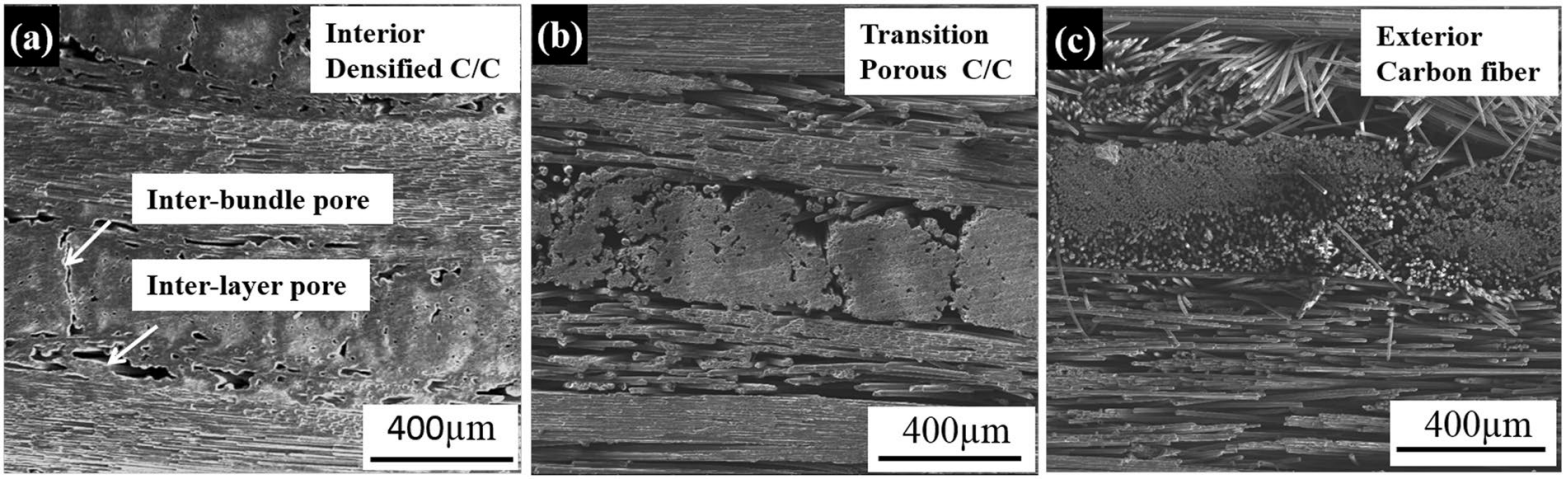
Typical microstructures of the green C/C in (**a**) interior region with a thickness of ~3 mm in the center of the composite, (**b**) transition regions between interior and exterior regions and (**c**) exterior regions with a thickness of ~1.5 mm in both two sides of the composite through-thickness direction.

Figure 1 shows the SEM morphologies of the green C/C in different regions fabricated by the first step E-CVI process. It can be found that a large amount of PyC matrix was deposited in the core region to form a dense C/C, also leaving a few inter-layer and inter-bundle pores (Fig. 1a). In the transition region, some PyC was deposited around carbon fibers to form a porous C/C, and a large amount of inter-layer and inter-bundle pores exist in this region compared with the core (Fig. 1b). However, almost no PyC was deposited in the exterior region and an original structure of the carbon fiber preform can be seen (Fig. 1c). The formation of the PyC gradient distribution is strongly dependent on the built-up of temperature gradient in the preform, initial deposition temperature and deposition time.

**Figure 2:**
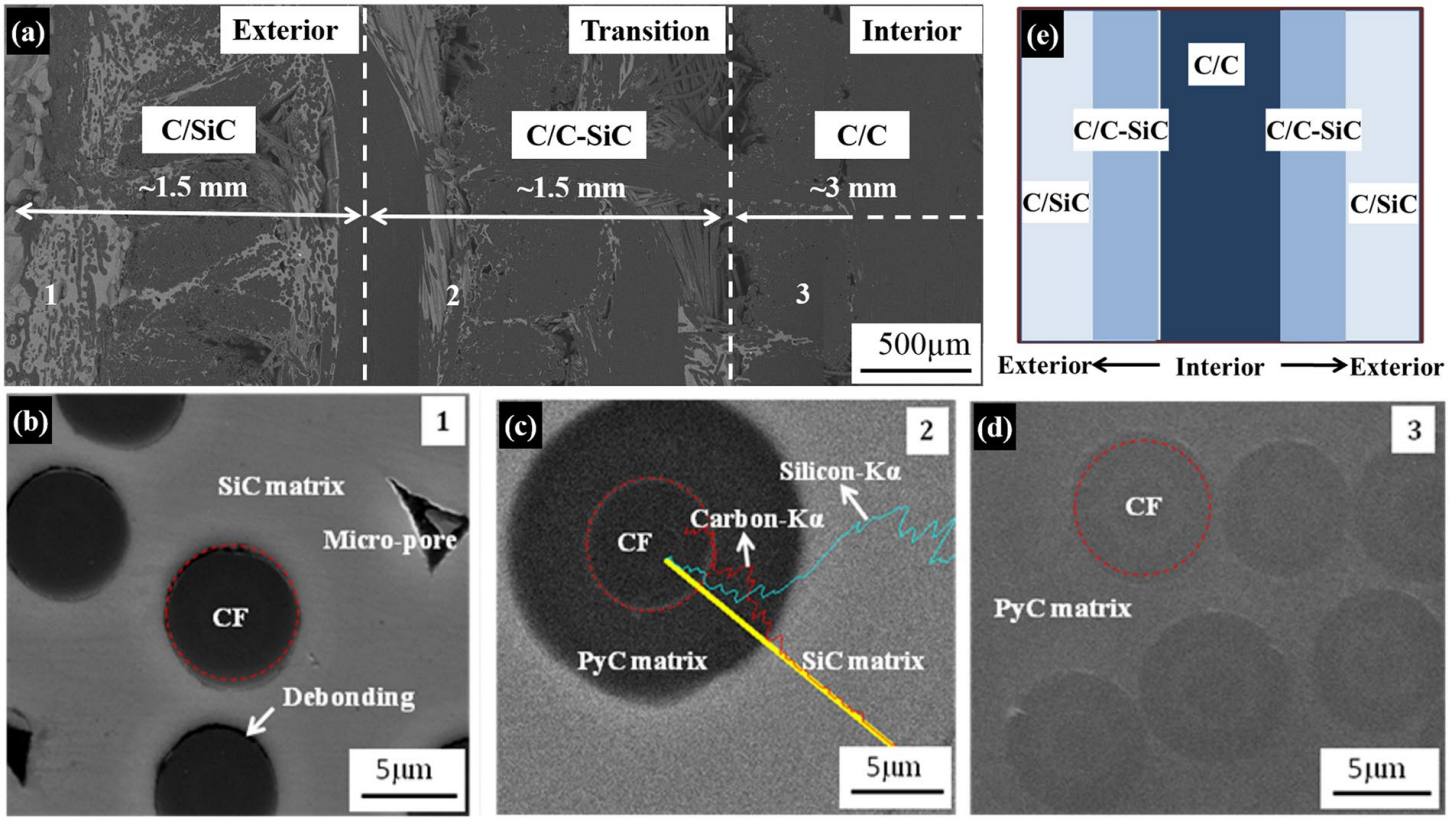
(**a**) Microstructure of the cross-section from exterior to interior region of as-prepared S-C/C-SiC composite, microstructures and EDS pattern of (**b**) area 1 in the exterior region, (**c**) area 2 in the transition region and (**d**) area 3 in the interior region, and (**e**) schematic diagram of the composite.

Figure 2 shows the cross-sectional microstructures and schematic diagram of the as-prepared S-C/C-SiC composite after the second step E-CVI process, and the typical microstructures in the three regions. It can be seen that the space in the porous region of the green C/C has been infiltrated by SiC, and even a thin SiC coating has been formed on the surface. The amount of SiC matrix displays a gradual increase from the core to the surface (Fig. 2a). The different regions in the S-C/C-SiC composite present the different microstructures and compositions since the two-step E-CVI process was used to deposit the PyC and SiC matrices. In the exterior regions, it is C/SiC composites since almost no PyC matrix was deposited in this region. The typical microstructure, just as position 1, is SiC matrix growth directly embracing around the carbon fibers, leaving some micro-pores between fibers. The interfaces between carbon fiber and SiC matrix are smooth and even some de-bonding can been found, indicating the weak interfacial adhesion (Fig. 2b). In the transition regions, it is C/C-SiC composite with gradient SiC matrix distribution. It can be seen that a distinct PyC phase is present on the surface of the fiber and then a thick SiC layer surrounds the PyC phase, and no interfacial debonding is found (Fig. 2c). In the core region, it is C/C composite with a small amount of SiC matrix (Fig. 2a,d). Finally, the C/C-SiC composite with a thickness of 9 mm exhibits a sandwich structure, containing two exterior C/SiC layers with about 1.5 mm thickness, two transitional C/C-SiC layers with approximately 1.5 mm thickness and an interior C/C core with 3 mm thickness (Fig. 2e).

To characterize the composition change of the green C/C and S-C/C-SiC composites along the thickness direction, the densities and volume fractions of different components in each layer were calculated, as shown in Table 1. Since the PyC is preferentially deposited in the interior of the preform during the E-CVI process, the density of the core with 3 mm thickness in the green C/C is 1.60 g/cm^3^, while those of the transition and exterior layers are 1.11 g/cm^3^ and 0.62 g/cm^3^, respectively. After the infiltration of SiC matrix, the densities of the two layers increase to 1.79 g/cm^3^ and 2.10 g/cm^3^ with SiC content of 21 vol.% and 46 vol.%, respectively. The final density of the S-C/C-SiC composite is 1.84 g/cm^3^.

**Table 1:** Densities in different layers and volume fraction of different components in the S-C/C-SiC composite.

Layer	Green C/C (g/cm^3^)	S-C/C-SiC (g/cm^3^)	Volume fraction of different composition in S-C/C-SiC (vol.%)			
			CF	PyC	SiC	Pore
Bulk (9 mm)	1.11 ± 0.02	1.84 ± 0.02	31	28	23	18
Core (3 mm)	1.60 ± 0.04	1.62 ± 0.05	31	52	1	16
Transition (1.5 mm)	1.11 ± 0.06	1.79 ± 0.05	31	28	21	20
Exterior (1.5 mm)	0.62 ± 0.03	2.10 ± 0.4	31	4	46	19

The sandwich structure and rapid matrix-deposition rate, different from the conventional CVI^14,15^, depend strongly on the unique deposition mechanism in the E-CVI process. The major features of the E-CVI process compared with the conventional CVI are the introduction of thermal gradient and electromagnetic fields into the deposition space of carbon fiber preforms. Firstly, a strong temperature gradient with higher temperature in the core of the preform is created due to the cold-wall effect and the low thermal conductivity of the preform. Therefore, the PyC deposits preferentially in the core region, and a structure with dense C/C in the core, partially or incompletely dense C/C in the transition layer, and porous fiber preform in the exterior layer can be achieved through controlling the temperature gradient and the deposition time. Secondly, the reactive gas containing MTS and H_2_ diffuses into the porous space of the green C/C composite to deposit SiC matrix, resulting in the formation of the S-C/C-SiC composites. The build-up of the temperature gradient is beneficial for the uniform mass transport of reactants from the surface to the interior, thus partially overcome the diffusion kinetics.

**Figure 3:**
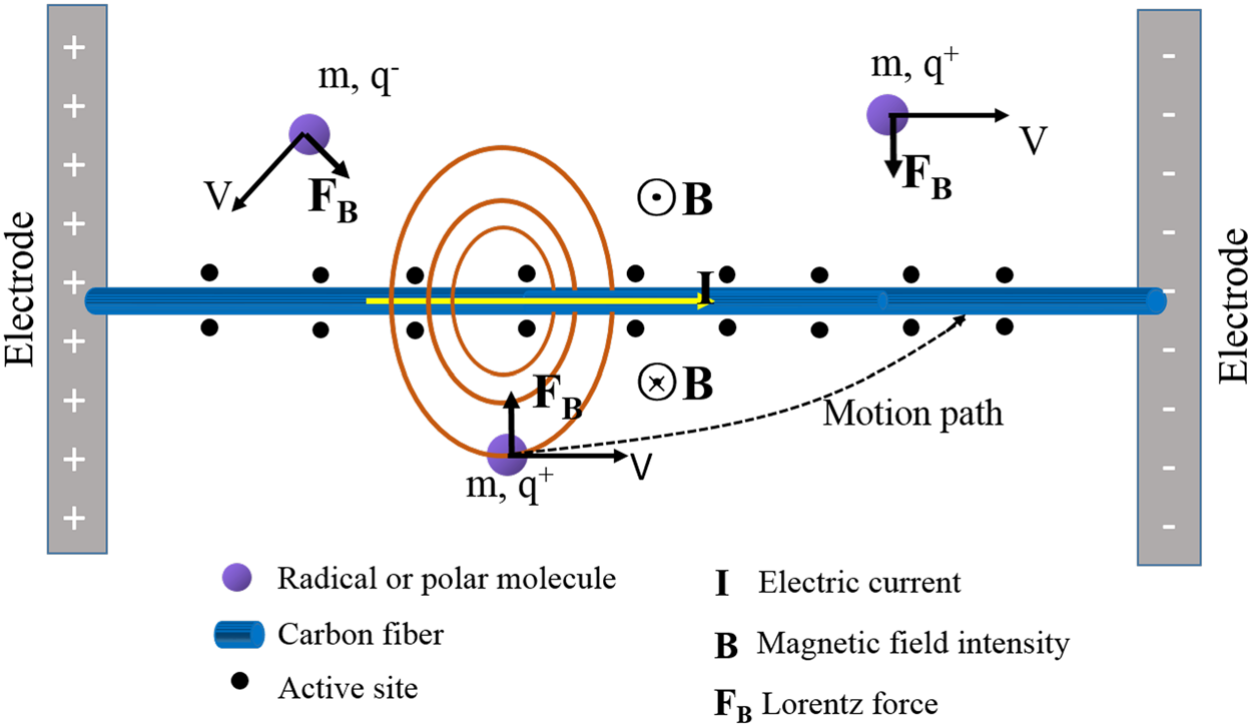
Schematic diagram of the electromagnetic coupling process.

To further elucidate the underlying mechanism of the rapid matrix-deposition rate, the two factors essentially improve the effective collision and the surface nucleation, as shown in Fig. 3. During the deposition of PyC and SiC in the E-CVI process, the active sites of fiber surface tend to greatly increase. The main reasons can be attributed to the three-fold facts: (*i*) the redistribution of surface charge, (*ii*) orientation re-construction of surface dipoles and (*iii*) change of surface defects (such as production of dangling bonds) when an electrical current was implemented on the fibers. In addition, the reactive intermediates such as radicals with unpaired electrons and polar molecules with permanent electric dipoles have inhomogeneous charge distribution in the molecular scale. The movement of the radicals and polar molecules toward the electrified fibers is accelerated. This can be understood briefly in two aspects based on the Biot-Savart law: one is that a fiber with current flow generates a concentric but non-uniform magnetic field, which tends to pull the radicals and polar molecules towards fiber by radially elevated field strength. The other is that the radicals and polar molecules with separated positive and negative charge centers usually experience a net magnetic force towards fiber with a force strength proportional to the fiber current, charge size, and inverse distance from the fiber. As a result, the possibility increases significantly for the effective collision and the chemisorption of radicals and polar molecules occurring on the active sites.

### SiC growth characteristics

**Figure 4:**
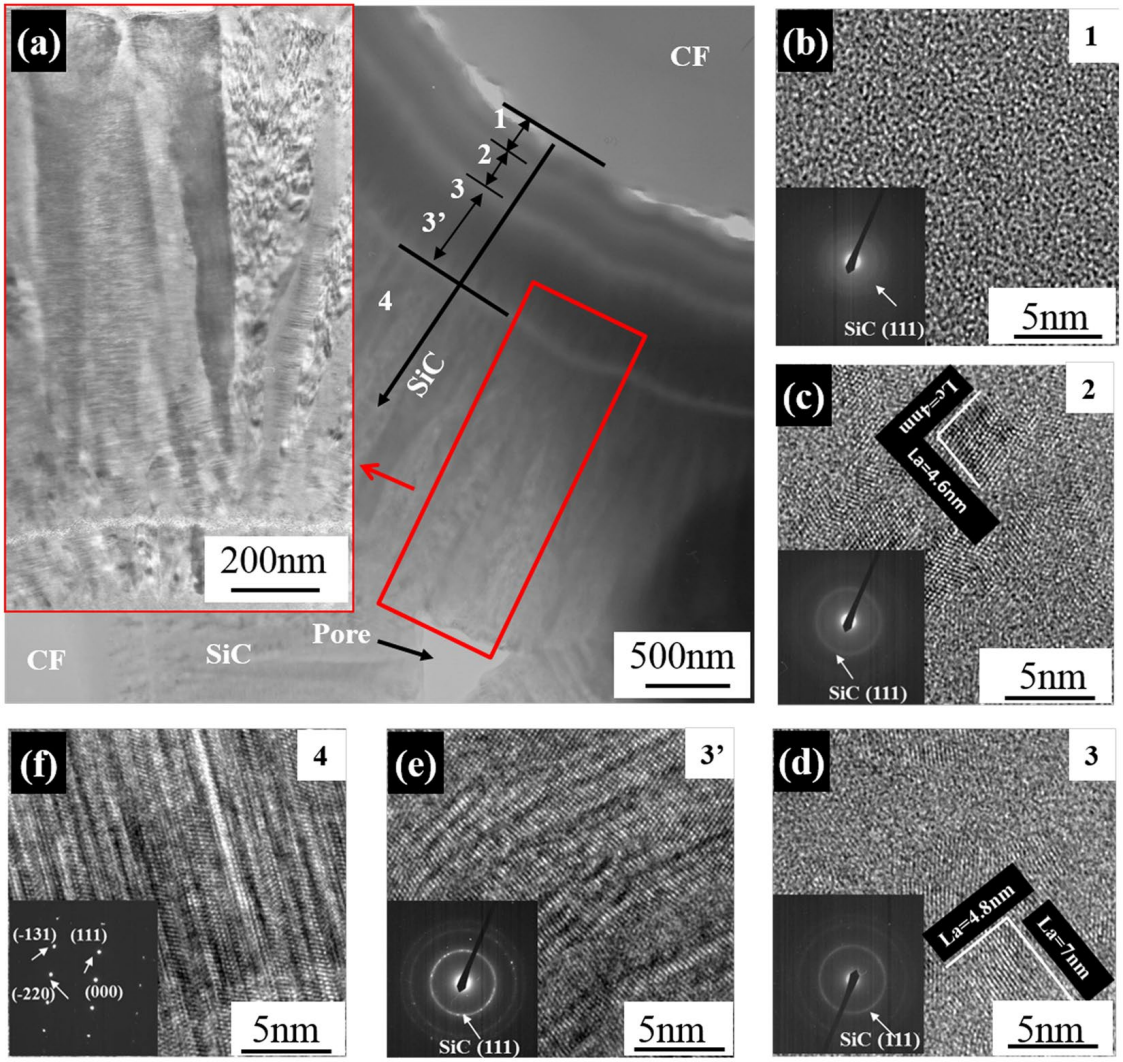
Bright-field TEM and HRTEM images of the S-C/C-SiC composites and the corresponding electron diffraction patterns: (**a**) CF-SiC interface and SiC matrix, (**b**) area 1, (**c**) area 2, (**d**) area 3, (**e**) area 3′ and (**f**) area 4.

**Figure 5:**
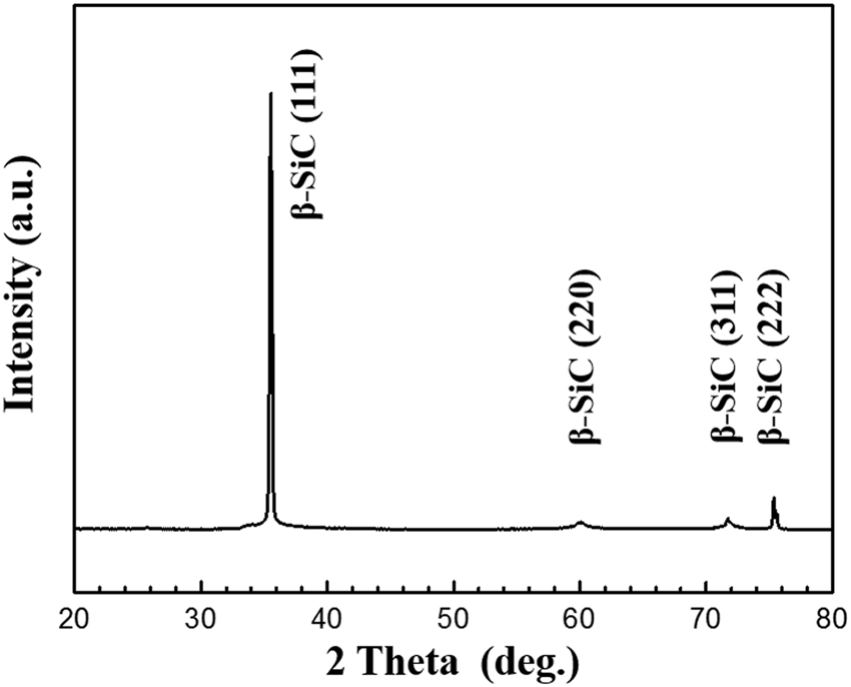
XRD pattern of the SiC matrix.

To further understand the growth characteristics of SiC matrix by the E-CVI, typical bright-field TEM and high resolution TE.

(HRTEM) images of the CF-SiC interface and SiC matrix and the corresponding selected area electron diffraction (SAED) patterns in the exterior layer of S-C/C-SiC composite are shown in Fig. 4. It can be seen that the SiC matrix grows surrounding carbon fibers to fill the space among the fibers, leaving some nano-scale pores (Fig. 4a). Between the SiC matrix and carbon fibers, tortuous interfaces exist. Some gaps (white color) with width ranging from several to dozens of nanometers are found along the fiber boundaries. The gaps were also found in the SEM image (Fig. 2b). In addition, the SiC matrix growth is discontinuous with blocking-up by several stripes in a width of dozens of nanometers. The inner part close to the fiber has a stratification structure of a width of approximatly 1 μm, indicating a slow growth rate. However, the outer part far from the fibers shows a dendritic feature, indicating a rapid crystal growth (Fig. 4a)^20–22^. Examination of the innermost layer marked by area 1 reveals the growth to commence with an amorphous SiC layer evidenced by the HRTEM image without crystalline structure and the SAED pattern without obvious diffraction rings (Fig. 4b). The region can be defined as an extended nucleation zone. Subsequently, some crystalline SiC clusters with a size of several nanometers embedded in the amorphous SiC appear in area 2 and 3; and the degree of crystallinity is enhanced slightly (Fig. 4c and d). In area 3′, the SiC is almost completely transformed into crystals with large size, accompanied by the appearance of bright and sharp SiC (111) ring with some discontinuous spots (Fig. 4e). The grow-up of the nano-crystals from area 2 to 3′ can be deemed as a competitive growth process. In area 4, the aggregate consisting of a large number of highly crystallized SiC grows approximately perpendicular to the stripe, and exhibits a dendritic feature with large columnar grains (Fig. 4a and f). The XRD pattern can indicate that the grains are the cubic SiC crystallographic phase with a pronounced preferred growth orientation in the (111) plane (Fig. 5).

**Figure 6:**
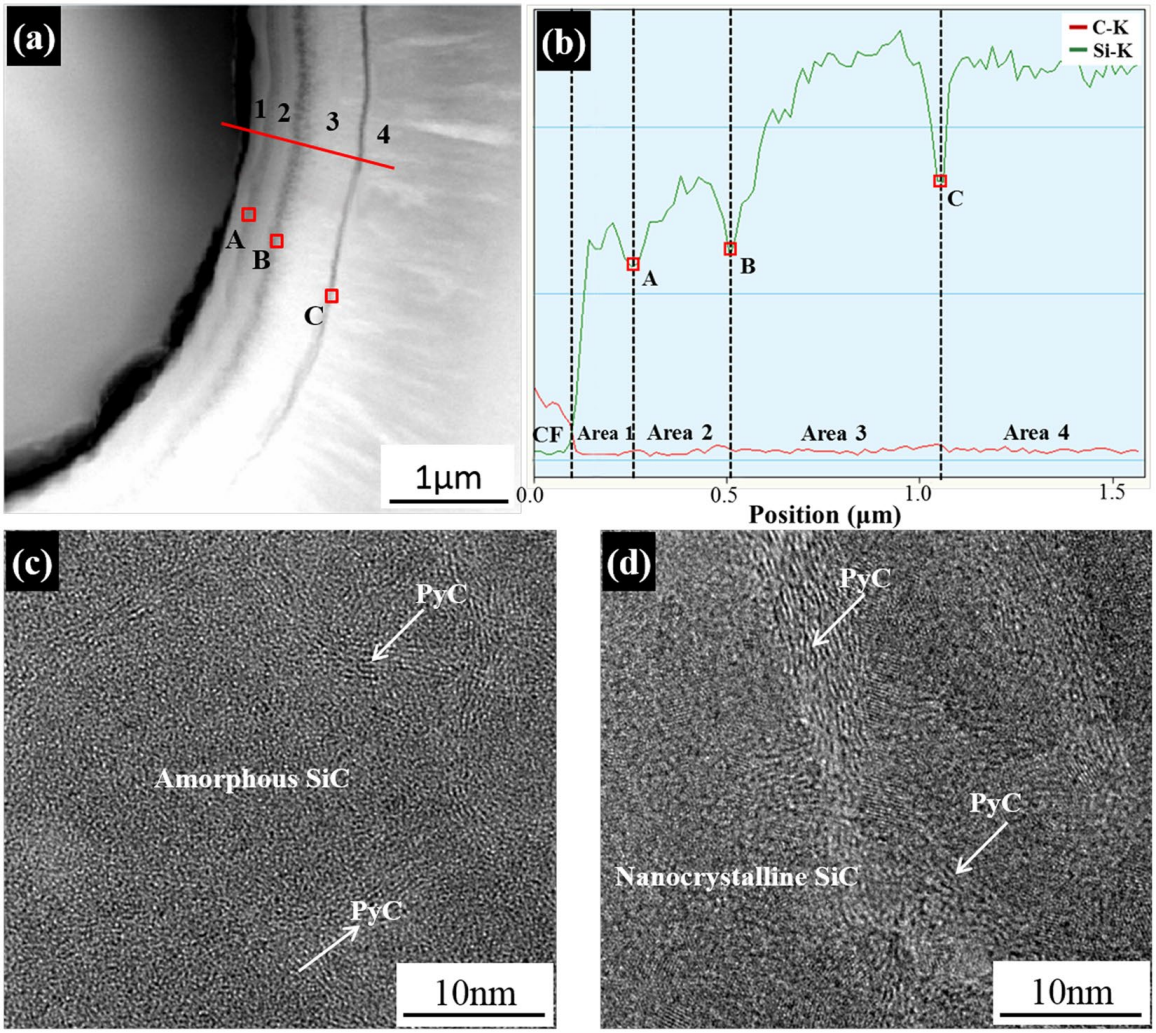
(**a**,**b**) HAADF-STEM image of stripes and its EDS line profile in the S-C/C-SiC composites, and (**c**,**d**) HRTEM images of interface A and interface B, respectively (interface C has a similar structure, not shown here).

In order to identify the composition of the stripes, high-angle annular dark-field STEM (HAADF-STEM) with EDS and HRTEM images were conducted, as shown in Fig. 6. In the stratification structure region, there is a tendency of the increasing Si content from the fiber surface to the matrix. In each stripe (marked by A, B and C in Fig. 6a), the Si content significantly drops while the C content raises (Fig. 6b). The HRTEM images in Fig. 6c and d further confirm that the stripe is a combination of amorphous SiC or nano-crystalline SiC and turbostratic-stacking PyC.

We concern ourselves with the particular morphologies of the SiC matrix considering three specified processes. Firstly, the amorphous SiC formation is controlled by the surface kinetics. It is clear that the SiC deposition certainly occurs, once the temperature of the fiber surface reaches the SiC deposition threshold (T_d_) at the initial stage. Simultaneously, at such a low or slightly higher temperature above T_d_, the small radicals and molecules favorably form due to the moderate gas-phase reaction. These small groups are going to be nuclei because they collide with the fiber surface and stick to the active sites, and these absorbed groups are expected to exhibit a limited surface mobility^20,21^. The combination of these groups contributes to the continuous and uniform nucleation of new growth sites and inhibits the grain growth, thereby, being beneficial to the formation of amorphous SiC. Secondly, as the deposition proceeds, the temperature of its frontier continuously increases due to the decrease of the electric resistance (the formation of parallel circuits between fiber and SiC sheathing). As a consequence, the intensification of gas-phase reaction and surface reaction caused by the higher deposition temperature give rise to the gradual crystallization of SiC and the grow-up of crystal grain sizes. This process is indeed controlled by the surface kinetics and mass transport. Thirdly, the large columnar grains finally occur after the temperature is fully increased and this process is controlled by mass transport^23,24^. Within this stage, a rapid rate of the chemical reaction occurs and the supersaturation decreases, in turn, further resulting in an increase of crystal grain size (or aggregate size) according to the Gibbs-Thomson relation. Additionally, the higher deposition temperature also provides an energetic prerequisite to easily overcome the active energy of surface diffusion. In this situation, it promotes to the fusion among the aggregates and the growth of the SiC crystallites in the large columnar grains. In addition, the gradually increasing reaction temperature at the reactive frontier should also respond to the composition change of the stratification structure region. The increasing deposition temperature causes the residual Si–Cl and C–H bonds decreasing, thus, resulting in increasing Si content and the formation of the stoichiometric SiC aggregates eventually^25^.

In addition, we have found the growth discontinuity with the stripes as boundaries, evidencing the independence of the different growth processes. However, the formation of the strip inclusion has been, unexpectedly, found to have some excess carbon. The fact is difficult to be explained since the reactive system has a relatively low deposition temperature, a high reactant concentration, and a low dilute ratio of H_2_/MTS. Seemingly, these several conditions would suggest the possibility of the existence of excess Si, rather than excess carbon. An explanation would be attributed to the fact that the C-containing molecular groups are ready to be formed in the gas phase since the existence of HCl inhibits the production of silicon-containing species in the atmospheric furnace.

### Mechanical and ablation properties

**Figure 7:**
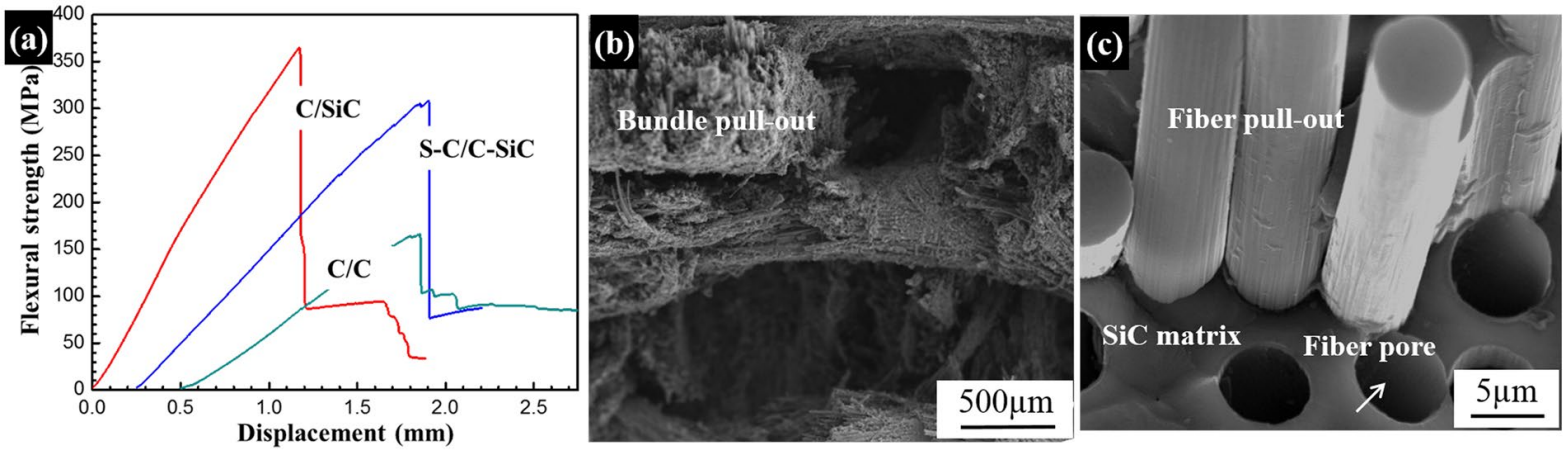
(**a**) Flexural stress-displacement curve and (**b**,**c**) fracture morphologies of the S-C/C-SiC composite.

Table 2 shows the density and strength of the as-prepared S-C/C-SiC composites and those of the C/C and C/SiC composites fabricated by the E-CVI process for comparison. It can be seen that the S-C/C-SiC composite exhibits good mechanical properties with a flexural strength of 325 MPa, which is 89% greater than the C/C fabricated by the E-CVI although the density of the S-C/C-SiC (1.84 g/cm^3^) is only a little higher than that of the C/C (1.75 g/cm^3^). Compared with the C/SiC with a flexural strength of 380 MPa (2.1 g/cm^3^), the S-C/C-SiC performs a comparable flexural strength in spite of a significant density reduction. The stress-displacement curves of these three composites exhibit non-linear fracture behaviors. The S-C/C–SiC composite possesses a medium modulus, a little higher than C/C and lower than C/SiC (Fig. 7a). It is well known that, when a composite suffers bending moments, the surface layer of one side is mainly subjected to tensile stress while the opposite to compressive stress, resulting in crack initiation and propagation in the initial loading phase. The feature of the surface layers in the composite may greatly affect the flexural properties^19^. In the S-C/C-SiC composites, there is a large amount of SiC matrix in the surface layer of two sides, which performs a composition similar to the C/SiC composites while different to the C/C composites.

**Table 2:** Comparison of the densities and strengths of the S-C/C-SiC, C/C and C/SiC composites.

	S-C/C-SiC	C/C	C/SiC
Density (g/cm^3^)	1.84 ± 0.02	1.75 ± 0.03	2.10 ± 0.05
Flexural strength (MPa)	325 ± 24	172 ± 14	380 ± 30

Compared with C matrix, the SiC matrix prepared by E-CVI has higher strength and modules, which can undertake larger bending moment until the initial cracks occur. Simultaneous, the cracks propagating in the SiC matrix need more fracture energy than in the C matrix. Thus, the S-C/C-SiC exhibits a higher or comparable flexural strength as compared to the C/C and C/SiC composites, respectively, and the good mechanical property is mainly attributed to the high strength and modules of the SiC matrix and the sandwich structure. From the fracture morphologies in the exterior layer, a jagged fracture surface with fiber bundle pull-out and an obvious de-cohesion between fiber and matrix leaving holes can be found (Fig. 7b and c). The fracture characteristics can be attributed to the existence of inter-bundle and inter-layer pores and the weak interfacial bonding between fiber and matrix. The carbon fiber has a radial and longitudinal CTE: 7.0 × 10^−6^(α_fr_) and -0.38 × 10^−6^ K^−1^(α_fl_), respectively. The CTE of SiC matrix is often taken as α_m_ = 4.8 × 10^−6^ ^26^. Since α_fr_ > α_m_, the carbon fiber generally contracts radically within the SiC matrix upon cooling with de-cohesion between the fiber and matrix (Fig. 4a). This interface characteristic possibly decreases the strength, but contributes to the toughness through interface de-bonding to arrest crack propagation.

**Figure 8:**
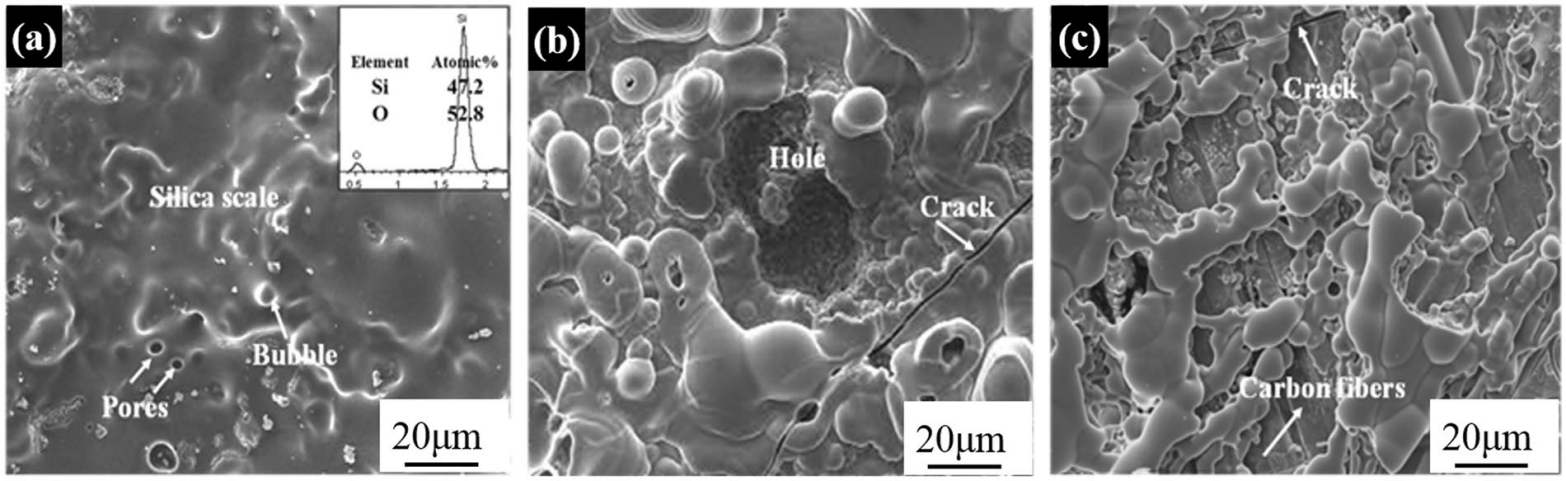
Morphologies and EDS pattern of the S-C/C-SiC composites after (**a**) one-cycle, (**b**) three-cycle and (**c**) five-cycle ablation.

As-expected, the S-C/C–SiC composite also presents good anti-ablation properties. The linear ablation rate is as low as 0.38 μm/s, close to that of C/SiC composite (0.34 μm/s), when exposing to 5-ablation cycles for 1000 s under an oxyacetylene flame test at ~1700 °C. Figure 8 shows the surface morphology evolution of the S-C/C-SiC after one-, three- and five-cycle ablation, respectively. After the one-cycle ablation, the surface is covered by an even and protective glass oxide layer without exposure of carbon fibers, which are proved to be SiO_2_ according to the atomic ratio of Si and O by the EDS pattern. A few bubbles and micro-size pores can also be found (Fig. 8a). After the three-cycle ablation, some ablation can be visible to the naked eye, and the ablated surface presents a discontinuous oxide film with some little pores, a hole of about a width of 20–40 μm, and even a penetrating crack (Fig. 8b). After the five-cycle ablation, the ablation is further aggravated with the severe breakage of the oxide scale, and the formation of a large pit of 380 μm in depth, a discrete SiO_2_ phase and many bare fibers (Fig. 8c).

## Conclusions

In this paper, we report the design and fabrication of a C/C-SiC composite by two-step E-CVI for a very short deposition time. Due to the sandwich structure design, the composite performs a low density, and a comparable mechanical and ablation property, compared to the C/SiC composites. In the E-CVI process, the SiC growth includes an extended nucleation zone with an amorphous SiC, a competitive growth region with a gradual crystallization of SiC and grow-up of nano-crystal, and a columnar grain growth region. The formation of thermal gradient in carbon fiber preform and the establishment of electromagnetic field around carbon fibers in the E-CVI process are responsible for the sandwich-structure formation, rapid matrix-deposition rate and SiC growth characteristics.

## References

[1] Odeshia AG, Muchab H, Wielageb B (2006). Manufacture and characterisation of a low cost carbon fibre reinforced C/SiC dual matrix composite. Carbon.

[2] Patel M, Saurabh K, Prasad V, Subrahmanyam J (2012). High temperature C/C–SiC composite by liquid silicon infiltration: a literature review. Bull. Mater. Sci..

[3] Lamouroux F, Bourrat X, Naslain R, Thebault J (1995). Silicon carbide infiltration of porous C-C composites for improving oxidation resistance. Carbon.

[4] Seifert H (2005). Yttrium silicate coatings on chemical vapor deposition-SiC-precoated C/C–SiC: thermodynamic assessment and high-temperature Investigation. J. Am. Ceram. Soc..

[5] Krenkel W, Berndt F (2005). C/C–SiC composites for space applications and advanced friction systems. Mater. Sci. Eng. A.

[6] Hald, H., Weihs, H., Reimer, T. & Ullmann, T. Development of hot CMC structures for space re-entry vehicles via flight experiments. AIAA/ICAS International Air and Space Symposium and Exposition: The Next 100 Y 14-17 July 2003, Dayton, Ohio, AIAA 2003-2696.

[7] Christin F (2002). Design, fabrication, and application of thermostructural composites (TSC) like C/C, C/SiC, and SiC/SiC composites. Adv. Eng. Mater..

[8] Tang SF, Hu CL (2017). Design, Preparation and properties of carbon fibers reinforced ultra-high temperature ceramic composites for aerospace applications: a review. J. Mater. Sci. Technol..

[9] Schulte-Fischedick J (2002). The morphology of silicon carbide in C/C-SiC composites. Mater. Sci. Eng. A.

[10] Li Y (2016). Strength evolution of cyclic loaded LSI-based C/C-SiC composites. Ceram. Int..

[11] Ly HQ, Taylor. R, Day. RJ (2001). Carbon fibre-reinforced CMCs by PCS infiltration. J. Mater. Sci..

[12] Kumar S, Bablu M, Ranjan A, Manocha LM, Prasad NE (2017). Fabrication of 2D C/C-SiC composites using PIP based hybrid process and investigation of mechanical properties degradation under cyclic heating. Ceram. Int..

[13] Suo J, Chen Z, Xiao J, Zheng W (2005). Influence of an initial hot-press processing step on the mechanical properties of 3D-C/SiC composites fabricated via PIP. Ceram. Int..

[14] Liu W, Wei Y, Deng J (1995). Carbon-fiber-reinforced C-SiC binary matrix composites. Carbon.

[15] Appiah KA, Wang ZL, Lackey WJ (2000). Characterization of interfaces in C fiber-reinforced laminated C–SiC matrix composites. Carbon.

[16] Tang SF, Zhou XM, Deng JY, Du HF, Liu WC (2005). The preparation of 2D C/C composites by chemical vapor infiltration. New Carbon Mater..

[17] Tang SF, Deng JY, Wang SJ, Liu WC (2007). Fabrication and characterization of C/SiC composites with large thickness, high density and near-stoichiometric matrix by heaterless chemical vapor infiltration. Mater. Sci. Eng. A.

[18] Deck CP, Khalifa HE, Sammuli B, Back CA (2013). Modeling Forced Flow Chemical Vapor Infiltration Fabrication of SiC-SiC Composites for Advanced Nuclear Reactors. Sci. Tech. Nucl. Install..

[19] Hu CL, Pang SY, Tang SF, Wang YC, Cheng H-M (2015). An integrated composite with a porous C_f_/C-ZrB_2_-SiC core between two compact outer layers of C_f_/C-ZrB_2_-SiC and C_f_/C-SiC. J. Eur. Ceram. Soc..

[20] Cheng DJ, Shyy WJ, Kuo DH, Hon MH (1987). Growth characteristics of CVD Beta-silicon carbide. J. Electrochem. Soc..

[21] Oh JH, Oh BJ, Choi DJ (2001). The effect of input gas ratio on the growth behavior of chemical vapor deposited SiC films. J. Mater. Sci..

[22] Shinozaki SS, Sato H (1978). Microstructure of SiC prepared by chemical vapor deposition. J. Am. Ceram. Soc..

[23] Buchannan FJ, Little JA (1991). The growth and morphology of chemically vapour deposited silicon carbide coatings on carbon-carbon composites. Surf. Coat. Technol..

[24] Lespiaux D, Langlais F, Naslain R, Schamm S, Sevely J (1995). Correlations between gas phase supersaturation, nucleation process and physico-chemical characteristics of silicon carbide deposited from Si-C-H-Cl system on silica substrates. J. Mater. Sci..

[25] Xu YD, Cheng LF, Zhang LT, Zhou W (1999). Morphology and growth mechanism of silicon carbide chemical vapor deposited at low temperatures and normal atmosphere. J. Mater. Sci..

[26] Xu YD, Cheng LF, Zhang LT, Yan DT (1998). Microstructure and mechanical properties of three-dimensional carbon/silicon carbide composites fabricated by chemical vapor infiltration. Carbon.

